# Myosin II sequences for *Lethocerusindicus*

**DOI:** 10.1007/s10974-017-9476-6

**Published:** 2017-07-13

**Authors:** Lanette Fee, Weili Lin, Feng Qiu, Robert J. Edwards

**Affiliations:** 1 Department of Cell Biology, Duke University, Box 3011, Durham, NC 27705 USA; 2 Shanghai Center for Bioinformation Technology, 1278 Keyuan Rd. Fl. 2, Shanghai, 201203 China

**Keywords:** Myosin II, Insect flight muscle, Alternative splicing, Interacting heads motif

## Abstract

We present the genomic and expressed myosin II sequences from the giant waterbug, *Lethocerus indicus*. The intron rich gene appears relatively ancient and contains six regions of mutually exclusive exons that are alternatively spliced. Alternatively spliced regions may be involved in the asymmetric myosin dimer structure known as the interacting heads motif, as well as stabilizing the interacting heads motif within the thick filament. A lack of negative charge in the myosin S2 domain may explain why *Lethocerus* thick filaments display a perpendicular interacting heads motif, rather than one folded back to contact S2, as is seen in other thick filament types such as those from tarantula.

**Electronic supplementary material:**

The online version of this article (doi:10.1007/s10974-017-9476-6) contains supplementary material, which is available to authorized users.

## Introduction

The indirect flight muscles of the giant waterbug, *Lethocerus spp*., have long held a special place in muscle research, thanks to their conveniently large fiber size and exquisitely well-ordered filament lattice. Key evidence that informed the early swinging crossbridge theory of contraction (Huxley and Brown 1967; Huxley 1969) was the observation of tilted myosin heads attached to actin in rigor *Lethocerus* muscle (Reedy et al. 1965). The first time-resolved X-ray diffraction of actively contracting muscle took advantage of the oscillatory contraction mode of *Lethocerus* muscle (Tregear and Miller 1969). The subsequent development of modern synchrotron X-ray sources was initially driven by the problem of muscle (Holmes and Rosenbaum 1998), and the first synchrotron X-ray pattern was recorded from *Lethocerus* muscle (Rosenbaum et al. 1971). The first cryo-EM images of isolated myosin filaments were also from *Lethocerus* (Menetret et al. 1988).

**Fig. 1:**
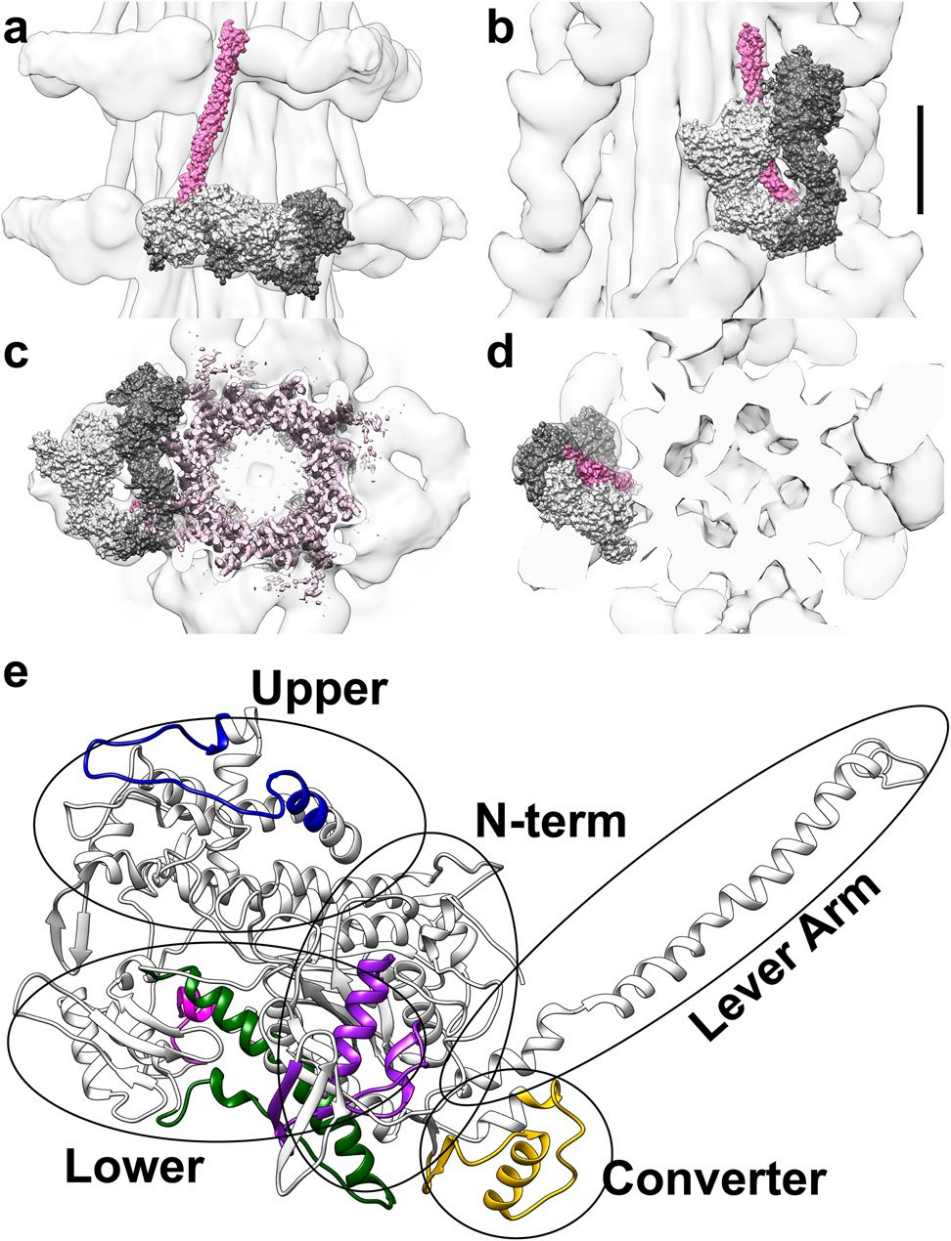
**a**–**d** Thick filament reconstructions from *Lethocerus* (Hu et al. 2016) on the *left* (**a, c**) and tarantula (Alamo et al. 2016) on the *right* (**b, d**) are shown in longitudinal (**a, b**) and cross-section views (**c, d**). A space-filling model of the myosin IHM, PDB 3JBH (Alamo et al. 2016), is fit within both maps. Although myosin is a dimer, the two heads of the IHM are not equivalent. One head (*light gray*) is called blocked, because its actin-binding domain contacts the back of its partner, called the free head (*dark gray*). In b and c, the IHMs are oriented similarly, although the filaments are perpendicular in these two views. Thus, the IHM is perpendicular to the S2 domain (*pink*) in *Lethocerus* (**a**); whereas it folds back to lie on top of the S2 domain in tarantula (**b**). The thick filament bare zone is towards the top of the page (**a, b**) or below the plane of the page (**c, d**). *Scale bar* 100 Å. **e** Ribbon diagram of myosin S1 head from the tarantula model, residues 1-838 of PDB 3JBH.g (Alamo et al. 2016), shows the five regions expected to be alternatively spliced in *Lethocerus*, color coded *purple, blue, dark green, magenta*, and *yellow* for MXEs 1, 5, 7, 8 and 10, respectively. A sixth alternatively spliced region is expected within the helical rod domain (not shown), near the junction between S2 and light meromyosin (LMM). The N-terminal, upper and lower 50 kD, converter, and lever arm domains of the S1 head are circled and labeled

A recent three-dimensional reconstruction of the myosin-containing thick filament from *Lethocerus* yielded several surprises (Hu et al. 2016). The myosin heads formed an interacting heads motif (IHM), which had previously been observed in smooth muscle myosin crystals (Wendt et al. 1999) and in other striated muscle thick filament types (Woodhead et al. 2005, 2013; Zoghbi et al. 2008; Zhao et al. 2009). But in the *Lethocerus* thick filament, the IHM was oriented in a unique way, perpendicular to the filament axis (Fig. 1a–d). Other insects with asynchronous flight muscle may have the same orientation of the IHM in the relaxed thick filament. Resolution in the latest reconstruction was especially good within the backbone of the thick filament (~5 Å), revealing the twists and turns of the α-helical coiled-coil rod in its native environment, as well as extra non-myosin densities embedded within the backbone (Hu et al. 2016). With this data we can begin to construct an atomic model of the entire 160-nm long myosin rod domain; in turn making it urgent that we obtain the correct amino acid sequence for *Lethocerus* myosin, which was previously unknown.

In many species, the myosin gene shows clusters of mutually exclusive exons (MXEs) that are alternatively spliced to give different protein isoforms (Bernstein et al. 1986; Wassenberg et al. 1987; George et al. 1989; Odronitz and Kollmar 2008; Kollmar and Hatje 2014). Evolutionary analysis shows eleven potential MXE clusters that code for specific regions of the molecule, ten within the S1 myosin head (Fig. 1e) and one within the helical rod domain (Odronitz and Kollmar 2008; Kollmar and Hatje 2014). The ancestral arthropod gene is predicted to be intron rich, with 42 exons that are typically short (Kollmar and Hatje 2014). The exon numbering and which MXE clusters are present vary among different taxa due to variable intron/exon loss. For example, the *Drosophila* myosin gene retains MXE clusters 1, 5, 7, 10 and 11, but has single exons for the remaining potential MXE clusters, whereas Hemiptera like *Lethocerus* retain the same five MXE clusters plus cluster 8 (Kollmar and Hatje 2014). Additionally, a short or long C-terminus is encoded by either inclusion or exclusion of the penultimate exon, which has an early stop codon (Bernstein et al. 1986; Odronitz and Kollmar 2008; Kollmar and Hatje 2014). The generally accepted view is that the alternative splicing fine-tunes the biophysical properties of myosin as needed for different muscle types (Bernstein and Milligan 1997). We propose here that the alternative splicing may also affect the stability of the IHM and the structural differences seen in thick filaments from different muscle types.

## Results

We sought the expressed myosin sequence by cloning *Lethocerus* cDNA and initially retrieved 52 partially overlapping clones (Supplemental Information, Methods). Using the partial clones to design new primers, we retrieved and report here eight unique full-length myosin clones, two unique partial clones, and the 5′ and 3′ untranslated regions, termed clones X1–X12 (GenBank Accession #s MF071206-MF071217). Simultaneously we initiated whole genome shotgun sequencing from *Lethocerus* DNA. Genome annotation is still in progress, but the scaffold containing the muscle myosin sequence has been identified and analyzed (GenBank Accession # MF078003).

**Fig. 2:**
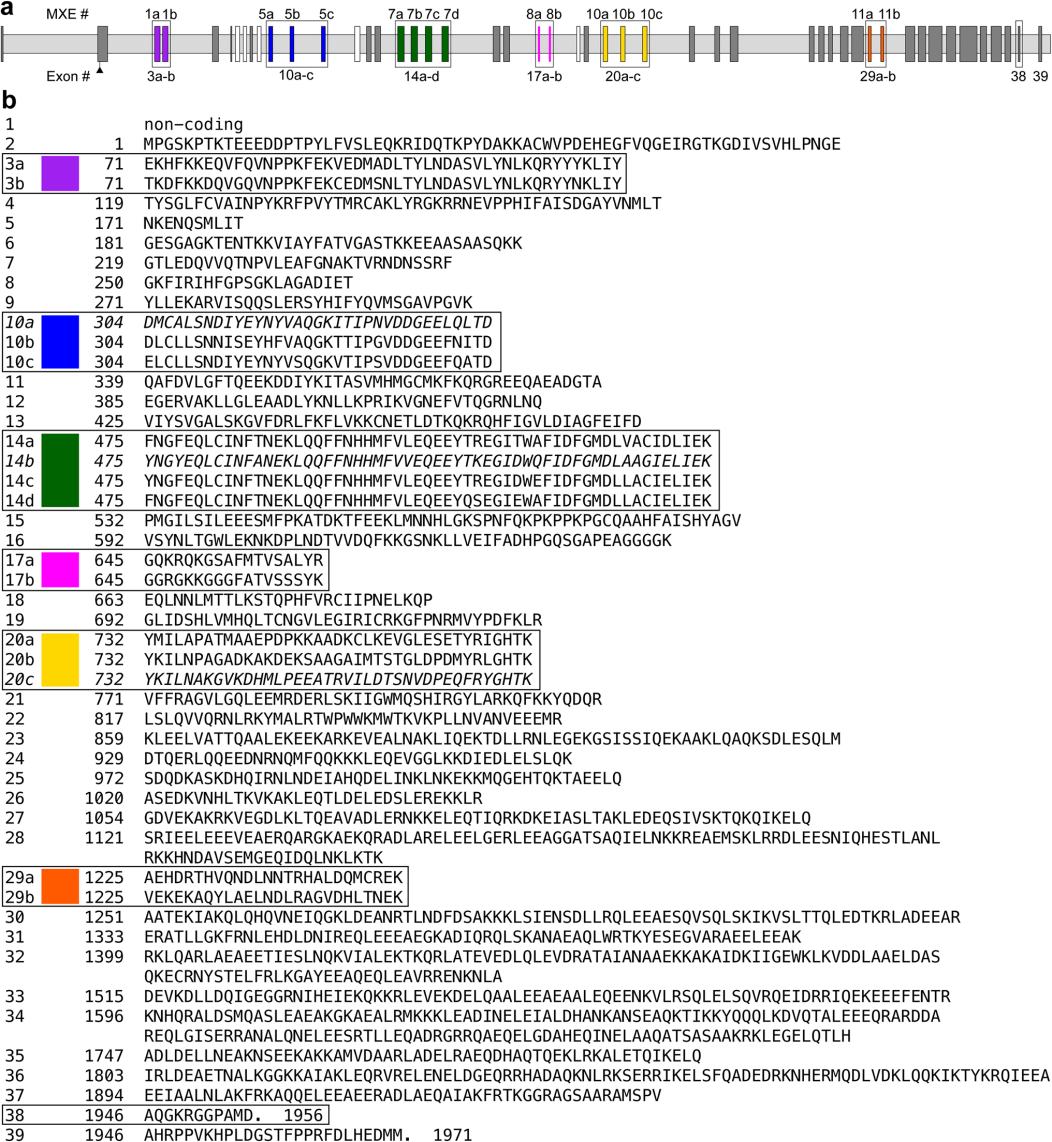
**a** In the *Lethocerus* myosin gene structure, *light gray bars* represent introns and *taller boxes* represent exons. *Numbers above* indicate the MXE cluster according to Kollmar and Hatje (2014); *numbers below* indicate the exon number within the *Lethocerus* myosin gene. *Dark gray boxes* represent single exons. MXE clusters 1, 5, 7, 8, 10 and 11 are outlined, numbered and color-coded. *Unnumbered white boxes* represent MXE clusters 2–4, 6 and 9, for which *Lethocerus* has single exons but some species have alternatively spliced clusters of MXEs. **b** The protein sequence is arranged by exon, with MXE clusters outlined and color-coded as in a. Exons 10a, 14b, and 20c are *italicized* to indicate they are putative exons for which no clone has been found to date

The gene structure and amino acid sequences of *Lethocerus* muscle myosin show the expected six MXE clusters that are alternatively spliced as well as the short/long C-termini (Fig. 2). The gene has 39 exons that are typically short, similar to other Hemiptera and in contrast to *Drosophila* myosin with only 19 exons that are necessarily longer (Kollmar and Hatje 2014). The location of 34 of the introns exactly match those predicted for the ancestral arthropod myosin gene [compare Fig. 2 to Fig. 5 of Odronitz and Kollmar (2008)], suggesting that the *Lethocerus* myosin gene is relatively ancient and undergone little intron loss. Similar to other species, the first exon lacks a start codon and is untranslated, and protein expression begins with the second exon (George et al. 1989; Odronitz and Kollmar 2008; Kollmar and Hatje 2014). At least one MEF2 promoter sequence and a number of E-box sequences are found either before the first exon or within the first intron, consistent with *Drosophila* myosin (Hess et al. 2007). We also identified a 3′ polyadenylation site that was 233 bases downstream of the stop codon in the last exon. In the genomic sequence, this site includes both upstream and downstream polyadenylation signals, 20 and 10 base pairs away respectively, as expected (Retelska et al. 2006). We found clones expressing almost all of the possible variants for each MXE cluster, with the exception of exons 10a, 14b, and 20c, which must still be considered putative variants (Fig. 2b, *italics*).

Our initial cloning used cDNA prepared from mixed muscle types, so we expected multiple clones. We were most interested in knowing which myosin isoforms are expressed in the dorsal longitudinal muscles, which are the ones usually used in muscle research. Therefore we prepared cDNA from mRNA separately isolated from the dorsal longitudinal, the oblique, and the dorsal ventral indirect flight muscles, and then used PCR to screen for the presence or absence of every exon variant in each of the three muscles (Supplemental Fig. 1). All three muscles tested negative for expression of exons 10a, 14b, and 20c, confirming our initial cloning that these variants are not expressed at detectable levels in adult flight muscles. Surprisingly, the dorsal ventral muscles tested positive for all of the remaining exon variants except 14c; therefore nine of the ten unique clones we obtained appear to be expressed in dorsal ventral muscles. The dorsal ventral muscles are difficult to dissect and it is possible that our sample was contaminated with other muscle types, such as direct flight, skeletal, or somatic muscles, which may explain the apparent multiplicity of variants expressed. In contrast, the dorsal longitudinal and the oblique muscles are easy to isolate, and they expressed only exons 3b, 10c, 14a, 17a/b, 20a and 29a, as well as both the short and long C-termini. Thus we found that only clones X1, X2 and X4 are expressed in the dorsal longitudinal and oblique flight muscles (Supplemental Information, Table 1).

When clone X2 is used to query the known *Drosophila melanogaster* myosin sequences in a BLAST search (Altschul et al. 1997), it is 83–85% similar to all *Drosophila* myosins, but most similar to isoform K, the adult flight-muscle specific isoform (Zhang and Bernstein 2001). Clone X1 is identical to X2 except for having the long C-terminus, rather than the short one. In *Drosophila* indirect flight muscle, there is evidence of sequential expression of a long C-terminal myosin early in sarcomere development and a short C-terminal myosin later (Orfanos and Sparrow 2013). In contrast, our results indicate concurrent expression of both the long and the short versions in *Lethocerus* flight muscle. Clone X4 is identical to X1, except for expressing exon 17a, instead of 17b. Exon 17 corresponds to MXE 8 and codes for a variable region of the myosin motor domain known as loop 2 (Kollmar and Hatje 2014). We used quantitative PCR to estimate the relative levels in the dorsal longitudinal muscles and found that exon 17b was expressed slightly more than exon 17a (mean ratio = 4 ± 2, from three cDNA samples independently prepared from different insects).

## Discussion

**Fig. 3:**
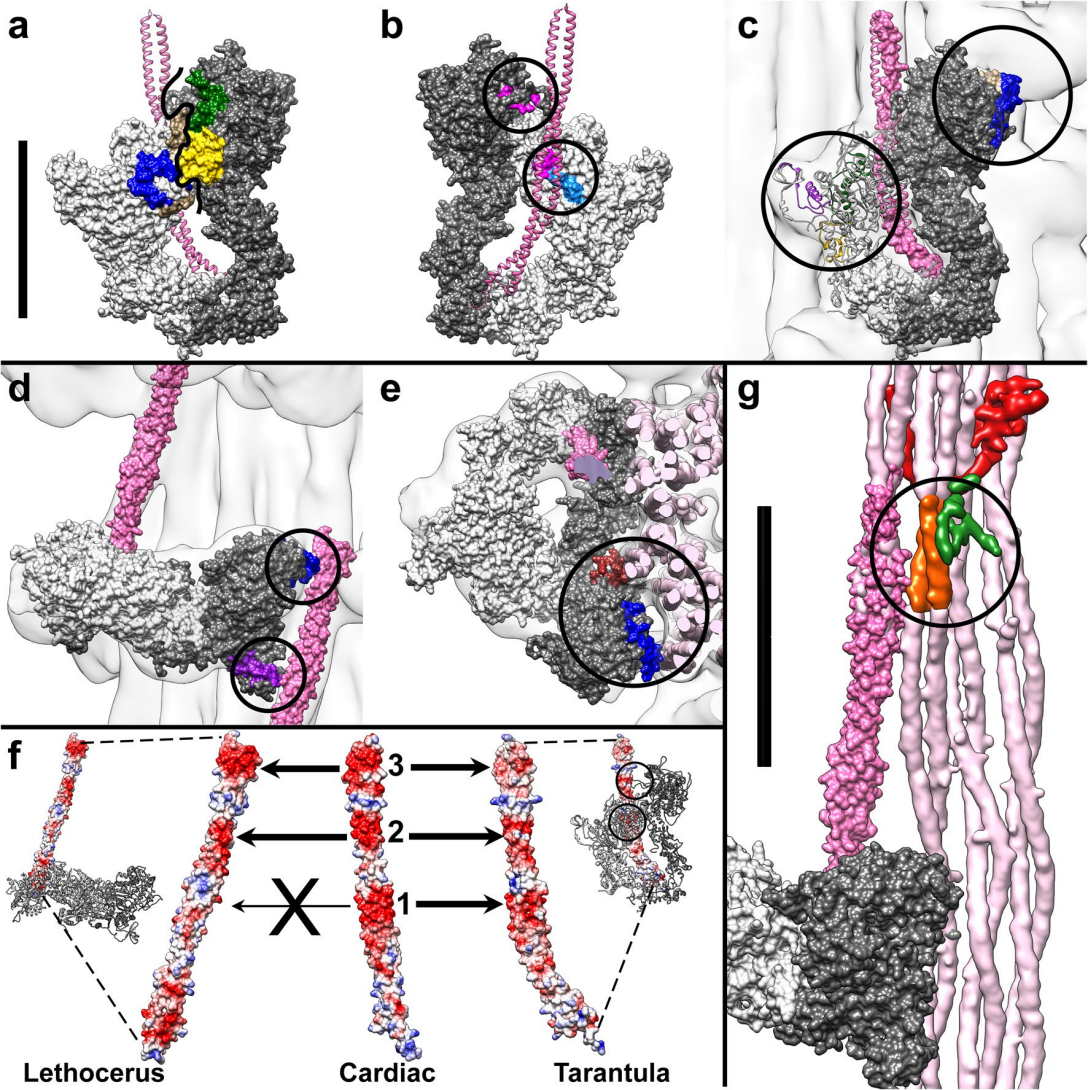
Color-coded MXEs are shown in context of the IHM and thick filament structures of tarantula (**a**–**c**) and *Lethocerus* (**d, e, g**). **a** Surface representation of the tarantula IHM shows blocked-head MXEs 5 and 6 (*blue* and *tan*) contacting free-head MXEs 7 and 10 (*green* and *yellow*). The *heavy black line* separates the blocked head (*light gray*) from the free head (*dark gray*). *Ribbon* representation of the S2 domain is shown in *pink* passing behind the IHM. **b** The view flipped 180° shows the back of the IHM where blocked-head MXEs 8 and 9 (*magenta* and *light blue*) contact the pink S2 domain (*lower circle*). In the upper circle, free-head MXE 8 (*magenta*) does not directly contact S2, but may be necessary to position the part that does contact S2. This region is a flexible loop known as loop 2 (Rayment et al. 1993). **c** Same view as **a**, with the transparent thick filament structure overlaid. In the *upper circle*, free-head MXEs 5 and 6 (*blue* and *tan*) contact an adjacent IHM. In the *lower circle* blocked-head MXEs 1, 7 and 10 (*purple, green* and *yellow*) contact the thick filament backbone. For clarity, the blocked head is shown as a ribbon diagram, whereas the free head is shown as a surface diagram. **d** In the *Lethocerus* IHM, free-head MXEs 1 and 5 (*purple* and *blue*) contact a neighboring S2 (*pink*). **e** A cross section shows the contacts of MXE 2 and 5 (*brown* and *blue*) to the thick filament backbone, where the α-helical coiled-coils can be seen (*light pink*). This view is flipped 180° relative to Fig. 1c. Because this is a rigid-body fitting of 3JBH to the *Lethocerus* thick filament structure, the three contacts shown here (**d, e**) should be considered speculative. In contrast, more confidence can be assigned to the tarantula contacts (**a**–**c**) because 3JBH was flexibly fit to the tarantula structure. MXE clusters 1, 5, 7, 10 and 11 (**a**–**e** *purple, blue, green* and *yellow*, and **g** *orange*) are alternatively spliced in both arachnids and *Lethocerus*; whereas clusters 2 and 9 (**e** *brown*, and **b** *light blue*) are alternatively spliced in arachnids but not *Lethocerus*, cluster 8 (**b** magenta) is alternatively spliced in *Lethocerus* but not arachnids, and cluster 6 (**a, c** *tan*) is not alternatively spliced in either but is in other species such as scallops (Kollmar and Hatje 2014). **f** N-terminal S2 structures from tarantula myosin (3JBH), cardiac myosin (2FXM), and *Lethocerus* myosin (homology model based on 2FXM) are color coded by electrostatic potential from negative (*red*) to neutral (*white*) to positive (*blue*). The *three red rings* of negative charge seen in the cardiac structure are numbered. *Lethocerus* myosin does not appear to have Ring 1. **g** In the *Lethocerus* structure, MXE 11 (*orange*) is within the thick filament backbone made of coiled-coil rod domains (*light pink*) and contacts two extra proteins (*green* and *red*) and the S2 domain (*dark pink*) of a neighboring molecule. Scalebars = 100 Å

To date, the most detailed model of the IHM is PDB 3JBH, which was flexibly fit into a 20-Å resolution map of the tarantula thick filament. 3JBH revealed conserved residues that may be involved in specific contacts that stabilize the IHM (Alamo et al. 2016). We note here that many of those proposed contacts involve the alternatively spliced MXEs as can be seen when the MXEs are mapped onto the IHM (Fig. 3). Intramolecular contacts between the two motor domains include blocked-head MXEs 5 and 6 with free-head MXEs 7 and 10 (Fig. 3a). Although we do not yet have a flexibly fit model of the *Lethocerus* IHM, similar contacts would be expected. Likewise, similar contacts would be expected when the IHM is observed in isolated myosin molecules (Jung et al. 2008). The head–head interactions of the IHM are thought to give rise to the very low fibrillar ATPase rate known as the super-relaxed state (Hooijman et al. 2011). We suggest that alternative splicing of the MXEs, in addition to its widely studied effect on acto-myosin interactions (Bernstein and Milligan 1997), may also affect the stability of the IHM, and therefore affect species-specific differences in the kinetics of ATP exchange in the super-relaxed state (Naber et al. 2011).

In the folded-back IHM structure seen in tarantula thick filaments, blocked-head MXEs 8 and 9 make an intramolecular contact with their own S2 tail (Fig. 3b). Additionally, free-head MXEs 5 and 6 are involved in intermolecular contacts with the adjacent IHM to the right and one level up, while blocked-head MXEs 1, 7 and 10 are involved in intermolecular contacts with the S2 arising from the IHM to the left and one level down (Fig. 3c). All of these interactions are totally absent or distinct in the *Lethocerus* thick filament structure. In the *Lethocerus* thick filament, only MXEs 1 and 5 contact the S2 domain coming from the IHM to the right and one level down (Fig. 3d), and MXE 2 contacts the thick filament backbone (Fig. 3e). In particular, MXE 8 is not positioned to make any potential contacts within the *Lethocerus* IHM, unlike the tarantula IHM in which blocked-head MXE 8 contacts S2 (Fig. 3b). This structural difference suggests some difference in S2 (and/or MXE 8, discussed later). A crystal structure of this portion of S2 from human cardiac myosin revealed three rings of concentrated negative charge, termed Rings 1–3. The contact between blocked-head MXE 8 and S2 seen in the tarantula structure involves Ring 1 (Blankenfeldt et al. 2006). We built a homology model of S2 using the *Lethocerus* sequence and it reveals that *Lethocerus* myosin has much less negative charge at Ring 1, compared to cardiac or tarantula S2 (Fig. 3f). Therefore, lacking the Ring 1 negative charge, *Lethocerus* myosin may be unable to stabilize the folded-back orientation and thus prefers the perpendicular orientation of the IHM.

Alternatively spliced MXE 11 is located within the α-helical coiled-coil rod domain of myosin, near what is known as the S2-LMM junction. The S2-LMM junction is susceptible to proteolysis and the location of a bend in the rod domain *in solubilized molecules*, and is therefore thought to act as a hinge (Elliott and Offer 1978; Suggs et al. 2007; Miller et al. 2009). However, the *Lethocerus* thick filament structure shows this site to be well embedded within the thick filament backbone (Hu et al. 2016) and unlikely to serve as a hinge in this context. MXE 11 is, nevertheless, the site of several interesting interactions (Fig. 3g). It makes contacts with two extra, presumably non-myosin, densities (Hu et al. 2016). It also contacts the S2 domain of the adjacent molecule where that molecule joins the backbone. Previous efforts to pull the entire length of S2 free from the thick filament backbone by swelling rigor muscles failed, and showed only the first 11 nm of S2 (Liu et al. 2006), indicating that the contact between S2 and MXE 11 must be fairly strong.

**Fig. 4:**
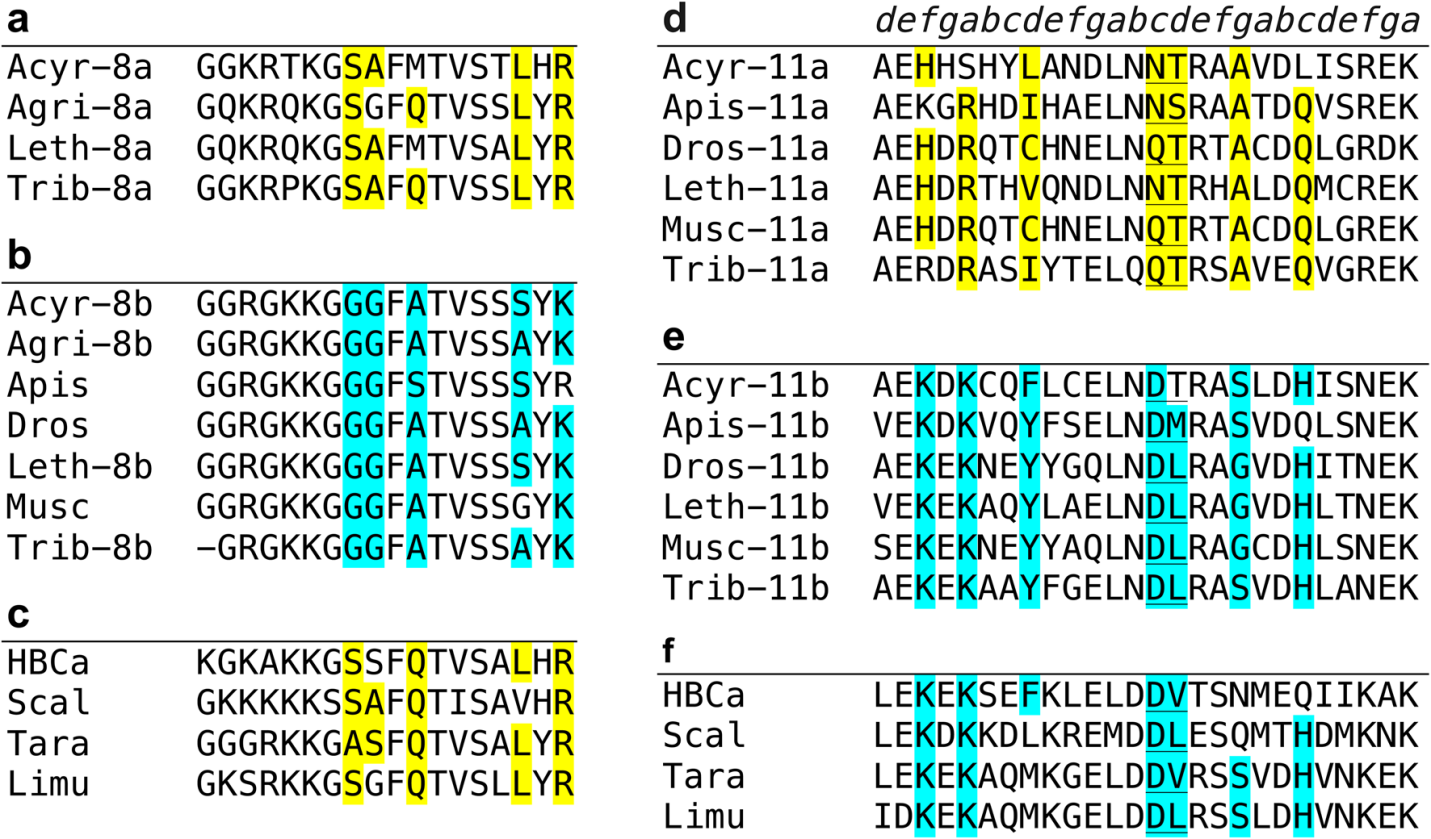
Sequence alignments of MXEs 8 and 11 show residues, highlighted in *yellow* and *cyan*, that are conserved but different in the two versions of each MXE. **a, b** MXE 8 sequences from seven insect species, *Acyrthosiphon pisum, Agrilus planipennis, Apis mellifera, Drosophila melanogaster, Lethocerus indicus, Musca domestica*, and *Tribolium castaneum*. **c** Sequences from human β-cardiac, scallop, tarantula, and *Limulus* myosins are more like the insect 8a versions. **d**–**f** In MXE 11, the sequences from human β-cardiac, scallop, tarantula, and *Limulus* myosins are more like the insect 11b versions. Most notable is a pair of residues at positions 14–15 (*underlined*). In the 11a versions this pair is polar and uncharged, asparagine or glutamine paired with serine or threonine. In contrast, in the 11b versions it is negatively charged aspartate paired with an aliphatic residue. The canonical heptad repeat for the α-helical coiled-coil is shown at the *top* of **d**

MXEs 8 and 11 typically have two variants if they are alternatively spliced, or a single exon otherwise (Odronitz and Kollmar 2008; Kollmar and Hatje 2014). *Lethocerus* dorsal longitudinal muscle expresses primarily MXE 8b. *Drosophila, Apis*, and *Musca* have a single exon for MXE 8, and they are more 8b-like when compared with insects that retain MXE 8 (Fig. 4a, b). All of these insects have asynchronous flight muscles, similar to *Lethocerus*. In contrast, sequences from human β-cardiac, scallop, tarantula, and *Limulus* myosins are more 8a-like (Fig. 4c). All four of these myosins have thick filaments with the folded-back IHM. Similarly, comparison of MXE 11 sequences shows that human β-cardiac, scallop, tarantula, and *Limulus* myosins are more 11b-like (Fig. 4d–f), in contrast to *Lethocerus* and *Drosophila* flight muscles which express MXE 11a (Miller et al. 2009; Suggs et al. 2007; Collier et al. 1990). Therefore, we suggest that this may be a general trend. Thick filaments will show the folded-back IHM if their myosin sequences in these regions are more similar to MXEs 8a and 11b, whereas they will show the perpendicular IHM if their sequences are more similar to 8b and 11a. This idea is complicated by our observation that *Lethocerus* dorsal longitudinal muscles express both MXE 8b and 8a. However, the *Lethocerus* IHM structure was based on a subset of thick filaments that excluded the filament ends and selected for perpendicular heads (Hu et al. 2016), so it is possible the reconstruction excluded heads with 8a sequence.

Further analysis awaits an atomic model of the rod and a flexible fitting of the *Lethocerus* IHM structure, which we are currently pursuing.

## Electronic supplementary material

Below is the link to the electronic supplementary material.

Supplementary material 1 (DOCX 1950 KB)
