# Supplementary material for: Myosin II sequences for Lethocerusindicus
Source: J Muscle Res Cell Motil. 2017 Jul 13;38(2):193–200. doi: 10.1007/s10974-017-9476-6 (PMC5660136; doi:10.1007/s10974-017-9476-6)
Supplement: Supplementary file 1 — Supplementary material 1 (DOCX 1950 KB) [file 10974_2017_9476_MOESM1_ESM.docx]

**Supplemental Information**

Myosin II sequences for *Lethocerus* indicus.

Journal of Muscle Research and Cell Motility

Lanette Fee^1^, Weili Lin^2^, Feng Qiu^2^, Robert J. Edwards^1^

^1^Department of Cell Biology, Duke University, Box 3011, Durham NC 27705, USA

^2^Shanghai Center for Bioinformation Technology, 1278 Keyuan Rd. Fl. 2, Shanghai 201203, China

Corresponding author: Robert Edwards, [rjpe@cellbio.duke.edu](mailto:rjpe@cellbio.duke.edu), 919-6845674

**Contents Page**

Title Page 1

Methods 2

Supplemental Figure S1 5

Supplemental Table 1 7

**Methods**

To clone the expressed myosin sequences, we used *Lethocerus* flight muscle cDNA, kindly provided by Dr. Belinda Bullard, with a polyC tail added using Terminal Transferase (NEB) and 100 uM dCTP (Bioline) as per the manufacturers protocol. To design cloning primers, we started with *Drosophila* myosin isoform K (Accession NP_724008.1) and used a protein BLAST search (Altschul et al. 1997) to identify homologs from *Acyrthosiphon pisum*, *Apis mellifera*, *Musca domestica*, and *Tribolium castaneum*, and their corresponding nucleotide sequences (Accession #s XM_008181012, XM_006569805, XM_011297437, and XM_008198304). These species represent the four major orders of insects with asynchronous flight muscle, similar to *Lethocerus* (Josephson et al. 2000). We used the regions of high homology among these sequences to design degenerate primers for the initial PCR round, along with a 13 nucleotide poly-G reverse primer. Initial amplification used OneTaq or Q5 polymerase followed by ligation into either pUC19 (Bam/HindIII) or pMiniT (PCR Cloning Kit, New England Biolabs), and yielded 52 overlapping partial clones covering the entire *Lethocerus* myosin heavy chain sequence, as well as a number of clones for paramyosin. For full-length myosin clones, we designed primers to the 5’ and 3’ ends, amplified with LongAmp Taq and TouchDown PCR (Korbie and Mattick 2008), and re-amplified with Q5 polymerase and standard PCR protocols. Full-length clones were inserted into pUC19 with restriction-free cloning (Bond and Naus 2012). Genomic sequencing was performed on an Illumina HiSeq 2500 platform.

For exon screening and real-time PCR, we prepared nine cDNA samples, one from each of the three major flight muscles (dorsal ventral, oblique, and dorsal longitudinal) from three separate *Lethocerus* *indicus* specimens. Flight muscles from a euthanized insect were either stored in RNAlater (ThermoFisher) prior to use, or directly homogenized in TRIzol (ThermoFisher). RNA was purified with a combined prep using TRIzol and RNeasy (Qiagen) (Untergasser 2008) and used to make cDNA with a Maxima H Minus Double cDNA synthesis kit (ThermoFisher).

We used the cDNA samples to screen for the expression of each of the alternatively spliced exons in the three flight muscles. Primers pairs were made to the variable portions of the exon in question and to a non-variable portion of an adjacent exon, except for exon 39. For exon 39, rather than making the second primer to exon 38 which can be variably included or excluded, the second primer was to exon 37. Primer pairs were expected to give PCR products of known length, from ~300-600 bp. The cDNA samples were then subjected to PCR using OneTaq polymerase and standard OneTaq PCR conditions. Primers to paramyosin were also included to serve as an internal positive control. PCR products were analyzed on a 1X TAE, 2% agarose gel stained with GelGreen (Biotium).

We used real-time qPCR to determine the relative ratio of exon 17b to 17a expressed in the dorsal longitudinal muscle. Primers were designed as above to give expected PCR products of 119 bp for exon 17a and 114 bp for exon 17b. Real-time qPCR was performed in quadruplicate and with 10-fold dilutions of starting cDNA from 1 – 10^-5^ on an Applied Biosystems StepOne Plus instrument using Power SYBR Green PCR Mastermix (ThermoFisher). The expression ratio was determined by

$$\frac{17b}{17a}=\frac{{(1+E_{17b})}^{-C_{T,17b}}}{{(1+E_{17a})}^{-C_{T,17a}}}$$

where the threshold concentration, C_T_, was determined by the Applied Biosystems software with ∆R_n_ set to 0.3 (confirmed to be in the exponential range for all samples), and the efficiency, E, for each reaction determined from the slope of the log-plot for the 10-fold dilutions.

For the figures, we built a chimeric homology model of the *Lethocerus* IHM (Fig 1 A, C and Fig 3 D-G) using the *Lethocerus* myosin heavy chain clone X1 sequence, residues 1-858, the *Lethocerus* regulatory light chain sequence (kindly provided by Dr. Belinda Bullard), and the essential light chain from Halyomopha halys (Accession # XP_014291721.1). These sequences were submitted to SWISS-MODEL (Biasini et al. 2014) and the structures predicted using the tarantula IHM (PDB 3JBH) as a template. This model was fit as rigid body into the *Lethocerus* thick filament structure (EM Databank #3301) as previously described (Hu et al. 2016). At the 20-Å resolution of the map in this region, the resulting fit of the tarantula-based IHM (from PDB 3JBH) was essentially identical to the previous smooth muscle-based IHM (PDB 1I84) used by Hu et al. (2016). Similarly, we fit a homology model of the *Lethocerus* N-terminal S2 domain (Fig 3 F-G), which was built using the *Lethocerus* sequence (residues 846-969) and the human cardiac S2∆ structure (PDB 2FXO) as a template. All 3D figures were generated using UCSF Chimera (Pettersen et al. 2004). The location of MXE 11 within the thick filament backbone (Fig 3 G) was predicted based on the contour length of the myosin rod domain, as previously described (Hu et al. 2016).

Altschul SF, Madden TL, Schaffer AA, Zhang J, Zhang Z, Miller W, Lipman DJ (1997) Gapped BLAST and PSI-BLAST: a new generation of protein database search programs. Nucleic Acids Res 25 (17):3389-3402. doi: 10.1093/nar/25.17.3389

Biasini M, Bienert S, Waterhouse A, Arnold K, Studer G, Schmidt T, Kiefer F, Gallo Cassarino T, Bertoni M, Bordoli L, Schwede T (2014) SWISS-MODEL: modelling protein tertiary and quaternary structure using evolutionary information. Nucleic Acids Res 42 (Web Server issue):W252-258. doi: 10.1093/nar/gku340

Bond SR, Naus CC (2012) RF-Cloning.org: an online tool for the design of restriction-free cloning projects. Nucleic Acids Res 40 (Web Server issue):W209-213. doi: 10.1093/nar/gks396

Hu Z, Taylor DW, Reedy MK, Edwards RJ, Taylor KA (2016) Structure of myosin filaments from relaxed Lethocerus flight muscle by cryo-EM at 6 A resolution. Science advances 2 (9):e1600058. doi: 10.1126/sciadv.1600058

Josephson RK, Malamud JG, Stokes DR (2000) Asynchronous muscle: a primer. J Exp Biol 203 (Pt 18):2713-2722

Korbie DJ, Mattick JS (2008) Touchdown PCR for increased specificity and sensitivity in PCR amplification. Nat Protoc 3 (9):1452-1456. doi: 10.1038/nprot.2008.133

Pettersen EF, Goddard TD, Huang CC, Couch GS, Greenblatt DM, Meng EC, Ferrin TE (2004) UCSF Chimera--a visualization system for exploratory research and analysis. Journal of computational chemistry 25 (13):1605-1612. doi: 10.1002/jcc.20084

Untergasser A (2008) RNAprep - Trizol combined with columns. Untergasser's Lab. <http://www.untergasser.de/lab/protocols/rna_prep_comb_trizol_v1_0.htm>. Accessed 6/22/2016 2017

**Supplemental Fig S1**

| **Exon** | **a** | **b** | **c** | **d** |
| --- | --- | --- | --- | --- |
| **3** | **408 DVM – –** | **328 DVM OM DLM** |  | |
|  | 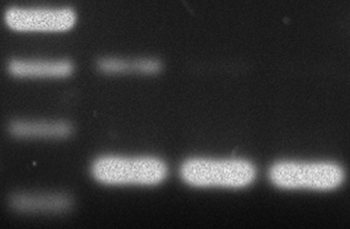 | 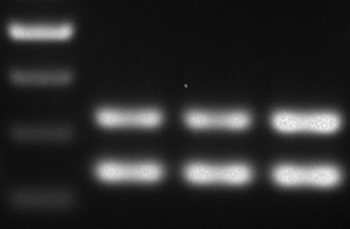 |  | |
| **10** | **517 – – –** | **298 DVM – –** | **329 DVM OM DLM** |  |
|  | 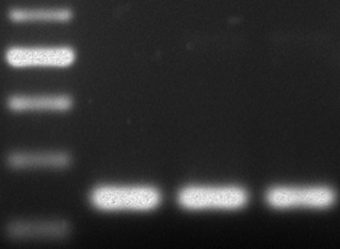 | 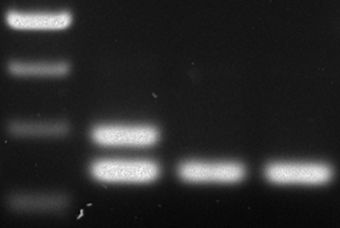 | 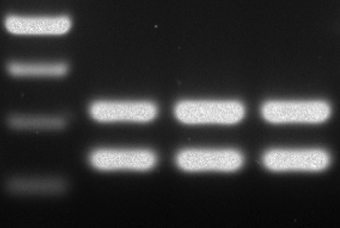 |  |
| **14** | **353 DVM OM DLM** | **335 – – –** | **336 – – –** | **352 DVM – –** |
|  | 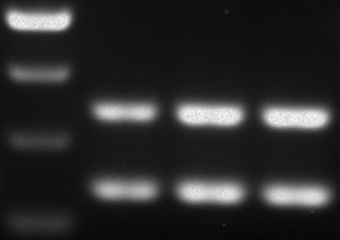 | 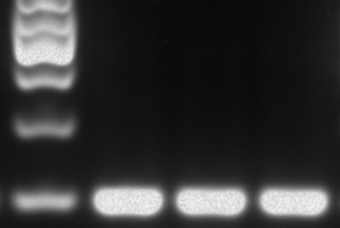 | 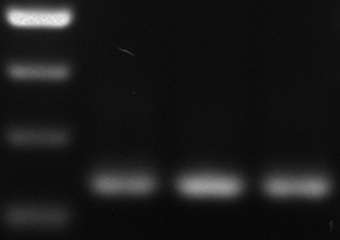 | 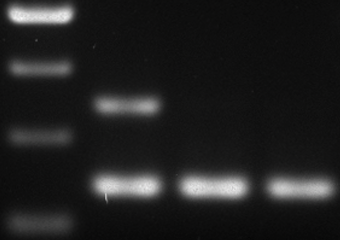 |
| **17** | **388 DVM OM DLM** | **453 DVM OM DLM** |  | |
|  | 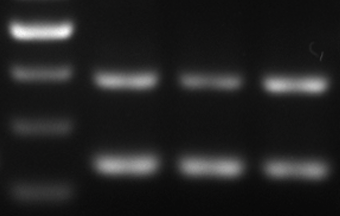 | 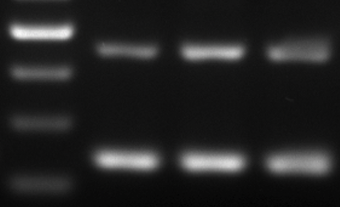 |  | |
| **20** | **406 DVM OM DLM** | **618 DVM – –** | **454 – – –** |  |
|  | 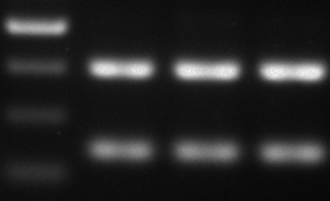 | 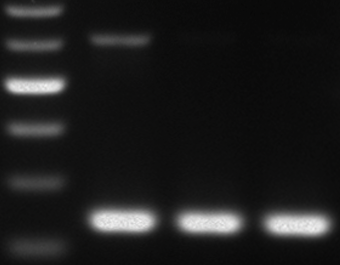 | 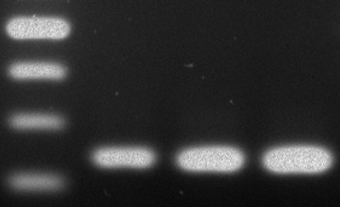 |  |
| **29** | **416 DVM OM DLM** | **390 DVM – –** |  | |
|  | 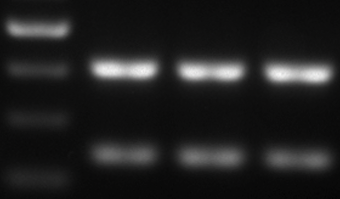 | 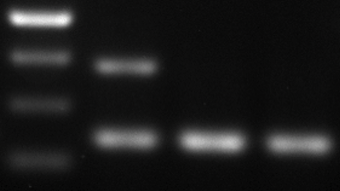 |  | |
| **38** | **360 DVM OM DLM** |  | | |
|  | 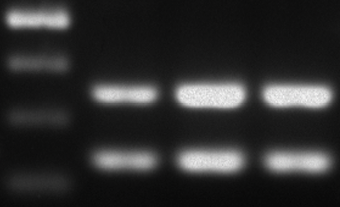 |  | | |
| **39** | **495**  **444 DVM OM DLM** |  | | |
|  | 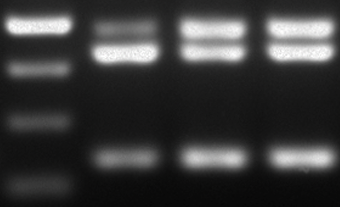 |  | | |

**Supplemental Fig S1** PCR screen of the alternatively spliced exon expression in the major flight muscles of *L.* *indicus*. For all gels, Lane 1 is a 100-kb ladder with the lowest band = 200 kb and the brightest band = 500 kb, Lane 2 is from the dorsal ventral muscle (DVM), Lane 3 is from the oblique muscle (OM), and Lane 4 is from the dorsal longitudinal muscle (DLM). In lanes 2-4, the three lower bands represent an internal positive control from primers specific for paramyosin with an expected size of 247 bp for all exons except 14b, which used a different set of paramyosin primers with an expected size of 194 bp. The upper bands in lanes 2-4, if present, indicate positive expression of the alternatively spliced myosin exon in question. The number above each ladder is the expected product size of the PCR test product for each exon, and labels above lanes 2-4 are the muscle type if positive or a minus sign if negative for the exon. Exon 39 shows doublets in all muscle types due to inclusion/exclusion of exon 38. The larger (upper) PCR product indicates the presence of mRNAs that also include exon 38, which codes for a short C-terminus due to an early stop codon. The smaller (lower) PCR product indicates the presence of mRNAs that exclude exon 38 and code for a longer C-terminus. Thus, the doublets in exon 39 indicated that all three flight muscles express both the short and the long C-terminus.

**Supplemental Table 1**

|  | **Exon** | | | | | | |
| --- | --- | --- | --- | --- | --- | --- | --- |
| **Clone** | **3** | **10** | **14** | **17** | **20** | **29** | **38** |
| **X1** | b | c | a | b | a | a | - |
| **X2** | b | c | a | b | a | a | + |
| **X3** | b | c | a | b | a | b | + |
| **X4** | b | c | a | a | a | a | - |
| **X5** | b | c | d | b | a | a | - |
| **X6** | b | c | a | a | a | b | - |
| **X7** | b | c | d | b | a | b | + |
| **X8** | a | b | a | a | a | a | + |
| **X9** |  |  |  | b | b | b |  |
| **X10** |  | b | c |  |  |  |  |

**Supplemental Table 1** Exon variants in each of the ten unique clones. Clones X9 and X10 are partial clones. Exon 38 is either included (+), resulting in a shorter C-terminus, or excluded (-), resulting in a longer C-terminus.
